# A Biological Inspired Cognitive Framework for Memory-Based Multi-Sensory Joint Attention in Human-Robot Interactive Tasks

**DOI:** 10.3389/fnbot.2021.648595

**Published:** 2021-11-23

**Authors:** Omar Eldardeer, Jonas Gonzalez-Billandon, Lukas Grasse, Matthew Tata, Francesco Rea

**Affiliations:** ^1^Dipartimento di Informatica, Bioingegneria, Robotica e Ingegneria dei Sistemi, Università di Genova, Genova, Italy; ^2^Robotics, Brain, and Cognitive Science Department, Istituto Italiano di Tecnologia, Genova, Italy; ^3^COgNiTive Architecture for Collaborative Technologies, Istituto Italiano di Tecnologia, Genova, Italy; ^4^Neuroscience/CCBN Department, The University of Lethbridge, Lethbridge, AB, Canada

**Keywords:** joint attention, multisensory integration, memory, decision-making, computational neuroscience, human robot interaction, active perception, biological motion control

## Abstract

One of the fundamental prerequisites for effective collaborations between interactive partners is the mutual sharing of the attentional focus on the same perceptual events. This is referred to as joint attention. In psychological, cognitive, and social sciences, its defining elements have been widely pinpointed. Also the field of human-robot interaction has extensively exploited joint attention which has been identified as a fundamental prerequisite for proficient human-robot collaborations. However, joint attention between robots and human partners is often encoded in prefixed robot behaviours that do not fully address the dynamics of interactive scenarios. We provide autonomous attentional behaviour for robotics based on a multi-sensory perception that robustly relocates the focus of attention on the same targets the human partner attends. Further, we investigated how such joint attention between a human and a robot partner improved with a new biologically-inspired memory-based attention component. We assessed the model with the humanoid robot iCub involved in performing a joint task with a human partner in a real-world unstructured scenario. The model showed a robust performance on capturing the stimulation, making a localisation decision in the right time frame, and then executing the right action. We then compared the attention performance of the robot against the human performance when stimulated from the same source across different modalities (audio-visual and audio only). The comparison showed that the model is behaving with temporal dynamics compatible with those of humans. This provides an effective solution for memory-based joint attention in real-world unstructured environments. Further, we analyzed the localisation performances (reaction time and accuracy), the results showed that the robot performed better in an audio-visual condition than an audio only condition. The performance of the robot in the audio-visual condition was relatively comparable with the behaviour of the human participants whereas it was less efficient in audio-only localisation. After a detailed analysis of the internal components of the architecture, we conclude that the differences in performance are due to egonoise which significantly affects the audio-only localisation performance.

## 1. Introduction

Robots approach a stage of technological advancement at which they will become a frequent partner in our daily lives. At this stage they regularly interact and engage in collaborative tasks with us. Humans and robots have to coordinate their actions in a shared environment in order to efficiently collaborate in these diverse scenarios. While humans are good at coordinating perception and action planning with their movements to achieve a common goal, such complex coordination is still an open challenge in robotics. When we collaborate with another human partner we recruit typical perceptual and action coordination skills. One of the most important coordination skills we use is joint attention as a fundamental mechanism to coordinate our actions (Schnier et al., 2011).

Joint attention can be defined as a shared attentional focus on the same perceptual events between multiple individuals (Reddy, 2005). It is used to coordinate between each of the agents toward a common object or event. Thus joint attention occurs as an emergent condition when a salient event captures the attention of both partners without a priori negotiation of the attentive target. For example, when two people are discussing a painting they are jointly seeing, the shared perception of the same painting allows them to exchange information about the same object. Joint attention is a natural phenomenon that we experience every day and can be triggered by different means: environmental-based (e.g., the appearance of a visual-auditory salient object in the environment) and social-based (e.g., eye-gazing, pointing, or other verbal or non-verbal indications) events (Mundy and Acra, 2006). Mastering correct joint attention with a partner is an important skill that facilitates collaborative interactions. It allows us to share our focus with another partner, enabling us to reason on a common basis. However joint attention not only must be correctly shared between interactants, but the timing of the focus shift also has to be comparable between the human and robot. Jointly shifting attention to the correct location is not necessarily useful if the timing fails to match human timing, as the interaction will fall out-of-sync. Joint attention has been studied extensively in humans, for its role in the development of children (Moore et al., 2014), in language acquisition (Tomasello and Farrar, 1986) and also as a way to identify autism (Bruinsma et al., 2004). Most of the studies on joint attention have been carried out in controlled environments, due to its complex nature and the diversity of scenarios under which it can occur. Current studies in joint attention between a human and an artificial system have mostly focused either on the human or the artificial agent performance. The assessment of combined performance (including mutual influence) across all the agents involved in the task is not common. A thorough assessment of both human attention and the attention of artificial agents would be relevant to the research community. In fact, research evidence shows frequently that both agents influence each other in joint collaborative tasks (Vannucci et al., 2017).

In cognitive architectures that take into account joint attention processes in order to create rich collaborative behaviours, other functionalities such as working memory might participate in attentional refocusing. Such components provide correct and accurate attention-timing and more importantly promote the intelligent behaviour of an attentive capable robotic agent. The influence that working memory has on the attentional mechanism is relevant (Mayer et al., 2007; Shipstead et al., 2014; Oberauer, 2019) but is rarely addressed in cognitive architectures for collaborative robots. Working memory has been defined as short-term memory used in order to proactively reinterpret the information in order to better operate in the environment (Miyake and Shah, 1999; Oberauer, 2019). Different computational models of attention for artificial agents have been proposed (Nagai et al., 2003; Triesch et al., 2006; Ognibene and Demiris, 2013) to respond to visual (Itti and Koch, 2001) and auditory stimuli (Treisman, 1996). However, these models do not fully consider the potential role of working memory related to the process of attentional focus redeployment. Some attention systems have been designed and evaluated to specifically address the context of collaboration between the human and the physically present robot partner (Admoni and Scassellati, 2017) but the potential role of memory remains only partially explored. In this work, we intend to endow the robot with the ability to rely on working memory, to reinterpret the information acquired in previous instances and states in order to better attend to the environment. Different possible computational models of working memory have been provided in different cognitive studies (Repovš and Baddeley, 2006) and in robotics applications (Phillips and Noelle, 2005). Inspired by these previous works,we provided the robot with a simple implementation of working memory that improves the attentive performance of the cognitive architecture for the humanoid robot iCub (Metta et al., 2008). The implementation engage the working memory component in a bio inspired decision making process.

Thus, we propose and evaluate the performance of a computational cognitive architecture for memory-based multi-sensory joint attention. Our goal with this study is to validate emergent joint attention guided by our cognitive framework. The architecture includes a multi-sensory attentional model, a working memory, a decision-making element, and an action executor (motor controller) to solve audio-visual stimuli localisation with human-like performance. We implemented a bio-inspired decision-making strategy (Murphy et al., 2016) for multi-sensory integration that will take into consideration both cognitive models of attention and the processing of working memory. We aimed at studying how the cognitive architecture responds in collaborative tasks between the iCub robot (Metta et al., 2008) and a human partner. We address the concept of joint attention emerging from a biologically-inspired multi-sensory selective attentional process defined as the selection of the relevant stimulus while ignoring irrelevant stimuli in the current environmental state (Nothdurft, 1991). With the goal of endowing an artificial agent with the ability to attend salient objects as humans do (accurate in location estimation and with optimal timing), we can promote emergent memory-based joint attention in collaborative scenarios. To evaluate the joint attention performance during unconstrained interaction and to exploit mutual influence between the parts, we compared human performance with the robot performance in a task in which both agents are exposed to the same salient audio or audio-visual stimuli. In particular, we focused on decision making as our main contribution, and we then addressed perceptual performance (localisation accuracy and reaction time) during the task. Our main testing and performance analysis is structured around three main hypotheses: H1-Memory-based Decision Making Process: The memory-based cognitive architecture is able to attend to multi-sensory stimulation and correctly take a decision based on the localisation process; H2-audio-visual vs. Audio only: The stimulus localisation accuracy and reaction time of the robot in audio visual task is better than in audio only tasks; H3-Robot Performance: The performance (accuracy and reaction time) of the robot will be as good as the performance of the human participants in localising the stimulus.

In section 2, we give a high-level description of the cognitive architecture and we describe the details of all the different components developed for each cognitive architecture layer (section 2.1). Then, section 2.2 describes the experimental design that tests the performance of the cognitive architecture. In section 3, we describe the results of the experimental session, and in section 4 we discuss the main results, drawing at the same time, some conclusions on the performance of the proposed cognitive architecture.

## 2. Materials and Methods

### 2.1. The Cognitive Architecture

**Figure 1:**
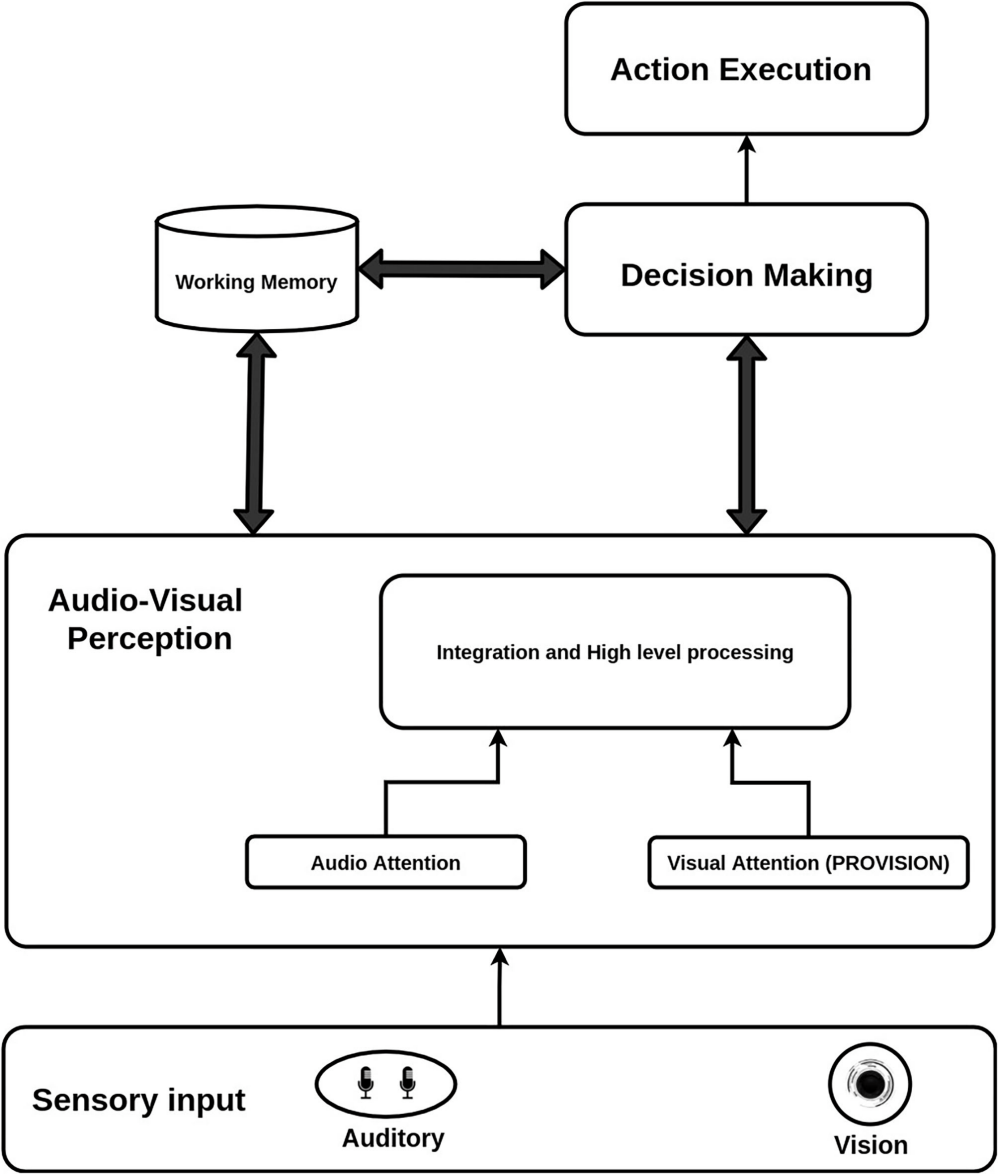
Over all cognitive architecture with all the main layers.

We designed the cognitive architecture (see Figure 1) with three main goals in mind. The first goal was to build a multi-modal (audio-visual) attention computational system to facilitate joint attention between a robot and a human during an interactive task. The second goal was to address the accuracy-time trade-off in decision making inspired by human behaviour. The third goal was to improve the attention, decision-making, and action execution cycle by including a working memory component. The first goal relates to the audio-visual perception component while, the second goal concerns the decision making process. Finally, the third one addresses the role of working memory in the decision making process The cognitive architecture is composed of four main building blocks. In this section, we will explain in details the four blocks (Audio-Visual Perception, Decision Making, Working Memory, and Action Execution). The details will include the biological inspiration, the overall process, and the connections between the different blocks.

**Figure 2:**
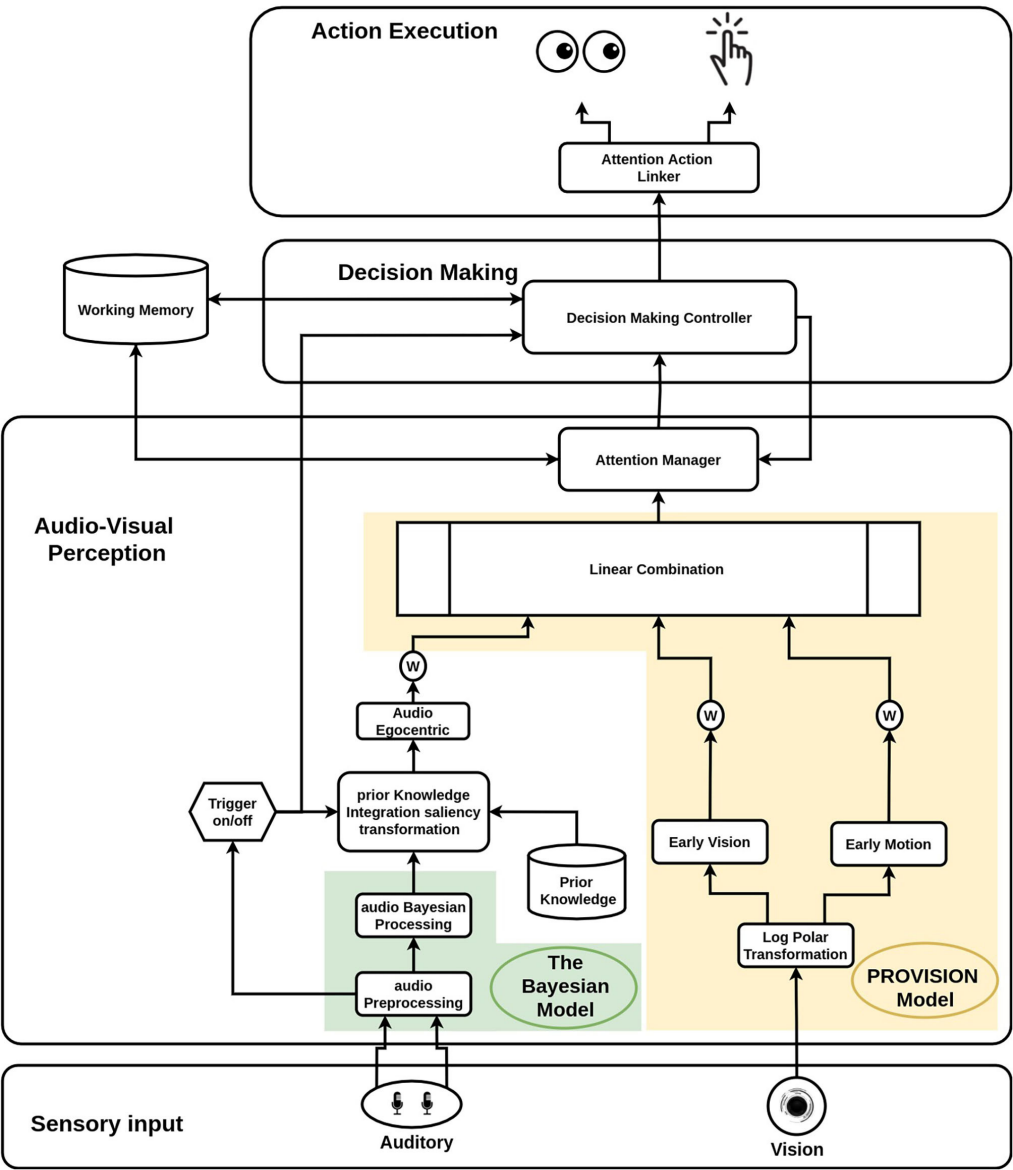
Detailed representation of the system implementation architecture.

The perception block uses early features from both of the sensory inputs (the audio and the vision) to trigger the start of the decision making process. The decision making process modulates perception to meet the task requirements and further sends commands to the motor control for action execution. Finally, the memory governs the entire process and is shared between all of the units. We will also explain the technical implementation for each component of the cognitive architecture after mentioned the overall functionalities of the component. Figure 2 outlines the structure and connections of our model's modules. Starting with the middleware, a software infrastructure that supports the integration of different cognitive modules, we used YARP Metta et al. (2010) (Yet Another Robot Platform) as our base. It is a multi-language middleware designed for robotic platforms. It is based on building multiple programs that run together in parallel and connect with peer-to-peer communication. We implemented our YARP modules using the C++ and python programming languages.

#### 2.1.1. Audio-Visual Perception

To facilitate attending to auditory stimulus, we built the audio attention component based on an existing bio-inspired Bayesian audio localisation model (Kothig et al., 2019). The auditory attention component redirects the attention of the robot toward salient auditory signals. The system is based on the biological basis of how humans perform sound localisation. Humans use different cues to localise sound sources: the interaural time difference (ITD) and interaural level difference (ILD). Both are differently recruited by the auditory system to derive the direction of sound arrival. In our implementation we focused on the ITD cue as the principal computational method since there is a robust literature that uses ITD for sound localisation in artificial systems (Argentieri et al., 2015). The general idea behind ITD is to infer the direction of a sound from the difference in time of arrival (TOA) between the two ears. Different approaches have been proposed in robotics to compute the TOA, the most common one is based on correlation metrics (Hosangadi, 2019). This approach performs well but is sensitive to noise and reverberation, which is problematic, especially in presence of ego noise produced by robots. Other biological systems in nature use ITD cues to localise sound by employing either banks of coincidence detectors connected by delay lines, as in the avian brainstem (Jeffress, 1948), or more complex phase-tuned mechanisms as in the mammalian brainstem (Grothe et al., 2010). The audio localisation model used in this research modelled the spectral decomposition of the human basilar membrane with a Gammatone filterbank and model delay-tuned units in the auditory pathway as banks of narrow-band delay-and-sum beamformers. To further deal with the spatial ambiguities associated with interaural cues (Blauert, 1997), the model uses a Bayesian regression model that infers the location of the sound source using the previous results of the spatial localisation values. As a result the location is reliably estimated in robot's allocentric coordinate frame as a probability distribution of sound source locations across azimuthal angles. This probability distribution is used to create an allocentric saliency map of the sound locations.

Another important aspect of attention is selective visual attention which allows an agent to focus on salient points in a visual scene. It acts as a filter, discarding non-essential information and retaining only important information for further higher cognitive processing. Itti and Koch (Itti and Koch, 2001) proposed a computational model of selective visual attention based on Treisman's (Treisman and Gelade, 1980) Feature Integration model of human visual attention. This model uses bottom-up flows of information, which are combined into a unified saliency map (Itti and Koch, 2001). In the Feature Integration model (Treisman and Gelade, 1980; Ruesch et al., 2008), the bottom-up information is processed to extract visual features such as edges, intensity, motion, and chrominance. High saliency within one of these low-visual feature maps allows the model to orient the focus of an agent toward salient points such as colourful objects, geometric forms, or moving objects. Following this idea, in this work, we used the PROVISION attention model developed for the iCub robotic platform (Rea et al., 2014). PROVISION is an implementation of the attention model of Itti and Koch for the robot iCub (Itti and Koch, 2001). It provides a modular tool for bottom-up attention, PROVISION integrates the different visual features with a weighted linear combination, enabling the ability to tune the importance of a particular visual stimuli, for example, forcing the attention toward a bright object by putting more weight on the intensity value. For the audio visual model, we had to implement an integration algorithm where both visual attention and audio attention are aligned and have the same representation. This integration is designed to be processed in the integration and high level processing component of the audio-visual perception block. In this component of the architecture the auditory attention is integrated together with the visual attention system. We remapped the allocentric auditory map into a visual egocentric saliency map. The map is then added as a feature to the linear combination of the attention system (already developed in the visual attention PROVISION model Rea et al., 2014). The sound then reinforces the visual saliency map at the corresponding azimuthal location only if the source of sound is located within the field of view. The aim of this process is to provide a unified multi-sensory saliency map which enables identification of salient points from both auditory and visual signals. After sensory integration, the output of the integration process is a saliency integrated map. Next, the saliency selection process happens, in which the system selects the point the model needs to attend to. As found in other attention models (Ognibene and Baldassare, 2015; Baldassarre et al., 2019), we moved from the cyclical selective attention systems which are typically used in robots to a temporally asynchronous method for selective attention. We therefore implemented the temporal asynchronous attention at salient changes in the landscape of the perceptual sensors. This allows the system to resemble the asynchronous attentional redeployment of humans. This selection is performed on the integrated scene and based on a time variant threshold which is defined based on a confidence-urgency trade off from the decision making block. When the selection process is finished, the selected point is then processed by the decision making block. This is where the Audio-Visual Perception block is connected to the decision making block. It is also connected to the working memory, in order to update the perceptual states in the memory for a better memory based decision making process. In this process a confidence-urgency trade off is performed based on the state time and the stimulation states. More details concerning the decision making block will be discussed in the following part (Decision Making).

Another added component in the audio-visual perception is the integration of prior knowledge for audio perception. The prior knowledge is the spatial locations of possible stimulation sources. This knowledge influences the perceptual abilities of the robot. This process is inspired by biological evidence about the importance of the prior knowledge in decreasing cognitive load, improving learning abilities, and improving perception (Cook, 2006; De Lange et al., 2018).

In Figure 2, the PROVISION model is highlighted with a yellow background colour and the audio Bayesian model is highlighted in a green background colour. The following part of this section is explaining in details the implementation of the added components to the audio-visual perception block which was mentioned above in brief.

##### 2.1.1.1. Trigger, and Prior Knowledge Integration

In order to overcome false positives coming from ambient sound in the environment, we integrated a power detection algorithm along with our sound localisation system as a relevant attentive mechanism in human audition (Rohl and Uppenkamp, 2012). We aimed to test the reliability of the sound power as an early informative feature. We added the calculations of the sound power in an early stage (audio prepossessing module) of the audio input. Using a fixed threshold on the total power for both audio channels, the system can determine whether the audio signal is high enough to be considered a valid sound or is just ambient noise. The threshold is autonomously extracted from the environment. The instantaneous sound power is used as an input to the trigger block. The trigger module receives the audio power processed by the audio prepossessing module. Based on a defined threshold for the instantaneous power, the trigger outputs signal to a higher level audio perception module (Prior Knowledge integration & saliency transformation) and also to the decision making block. Additionally, it updates the working memory which will be explained in a separate section.

Moving to the prior knowledge integration and saliency transformation module, we define two aspects of prior information for the audio stimulation. The first aspect is the possible locations of the stimulation. As the current audio system only considers the azimuth angle, this information is in a form of two lists. The first list is of angles describing where in azimuthal space the audio stimulation might be occurring and the second list is the spatial resolution of the angles, which reflects the size of the stimulation source. Thus for each stimulation source in the scene, we express the location in azimuthal allocentric angles from the robot's head axis as (X degrees ± resolution). These angles and their resolutions are the only locations that are considered from the allocentric probability map and the rest are ignored. The allocentric probability map is the output of the audio localisation model, which is a set of 360 values that represent the probability of the sound source's location at any arrival angle around the robot. These probabilistic values correspond to the 360 degrees centralised around the head axis. After considering the prior defined locations only, the resulting map is normalised to keep the Bayesian representation in the form of a probability distribution. By integrating this prior knowledge, we force the model to only focus on pre-biased defined locations. The second prior for the audio stimulation is the stimulation audio power. It is used to identify the threshold level of the sensitivity of the trigger. The trigger gives a high output if the audio power exceeded the threshold, which is the defined stimulation power level. Conversely, the trigger gives a low output if the audio power is less than this threshold. This signal is used to activate the transmission of the Bayesian map after adding the priors to the next stages. Otherwise, the transmitted map is a zero map. The trigger supports the prior knowledge module with the trigger signal to activate and deactivate the map transmission.

The next process is saliency transformation. The input of this process is the resultant Bayesian map after adding both priors (the stimulation activation level and the sources angles). The whole map is then multiplied by a total audio power and a scale factor. The audio power multiplication gives more importance to high stimulation than low stimulation (both are above the threshold level) and the scale factor transforms from Bayesian values (0–1) to the values of the monocular image (0–255).

##### 2.1.1.2. Audio Egocentric

The input of this module is an allocentric audio map, created after biasing the possible locations of audio sources. The allocentric map is 360 values for the 360 degrees of the azimuth plane. On the other hand, the visual attention system is egocentric with a retinotopic reference. The camera moves as well as the head of the robot, and based on these movements the robot sees different parts in the space. The aim of this module is to align the allocentric output of the spatial auditory system with the egocentric spatial vision. To integrate the audio to the visual attention system we had to perform this alignment. To achieve this task, the module needs to know the current state of the locations of both the head and camera in the azimuth direction. The process of extracting the egocentric map is based on the current locations on the camera and head in azimuthal direction and the camera parameters. The camera parameters specify the width of the area of vision, while the location states of the camera and the head specify the middle value in the area of vision range. Knowing the middle angular value and the angular width of the sound source, the module computes the starting and ending degree angles which then are extracted from the allocentric map. This is the first stage of the audio egocentric module which has an output of a subset from the allocentric saliency map of the audio. The second stage involves scaling these values vertically and horizontally to be in equal size with the frame size of the visual image. The horizontal scaling assumes that the audio source is from the horizontal level in the scene as we only consider the azimuth plane in the audio localisation module. The output of the scaling stage is now ready to be integrated as a feature in the PROVISION attention system with a defined weight in the linear combination part.

##### 2.1.1.3. Attention Manager

The attention manager is a central control module. It is responsible for analysing the combined scene from the output of the linear combination block of attention. The analysis is basically computing a confidence level. We propose a novel approach of recognising the unique target point of the scene to avoid continuous movement between different points. It is a measurement of the confidence level of uniqueness for the most salient point of the scene. We called this measure gamma value (Γ). The gamma value (Γ) represents how much the most salient point differs from the average salience across the scene. If the (Γ) value exceeds a threshold, then this point is identified as unique point of attentional interest. We call it a “hot point.” Γ is computed by calculating how far is the saliency of the maximum point from the triple of the standard deviation:


(1) $$\begin{array}{r} \Gamma =max\text{\_}value-mean\text{\_}value-3\sigma \end{array}$$

Where σ is the standard deviation of the combined saliency image. The Γ value gives information about the confidence level of uniqueness. Higher values are more likely to be a unique target whereas low values mean that in the scene there are multiple salient points with similar level of saliency. When a unique target is recognised [(Γ) value is greater than the current confidence threshold], it sends the selected point to the next connected elements in the architecture which is the decision making controller in the decision making block.

Additionally, the attention manager block receives manipulation commands for the threshold value from the decision-making layer. The threshold here represents the level of the confidence in which action is required. Therefore, the attention manager here can be presented as a trigger that acquires an action execution process for that current scene from the decision making block. Also, the module is able to fully control the process of suspending and resuming the attention process as well as the linear combination parameters. To summarise this part, the attention manager presents the main control unit of attention. It has the ability to change the attention parameter. It receives commands from other parts in the system, and finally it communicates with the other parts of the system and sends combined information about the current scene.

#### 2.1.2. Decision Making

From research theories elaborated on in the previous decade, visual processing in humans and animals triggers a decision-making mechanism in the form of a higher-level process, relying on the extraction of low-level features and properties from visual input (Vanrullen and Thorpe, 2001). This process is meant to evaluate the perceptual output properties and their relevance to the current goal and expectations.

Decision-making processes inspired by time-invariant models have been adopted for decades by the computational neuroscience community (Ratcliff and Smith, 2004). These models are based on a decision-making signal, which is triggered by a fixed threshold. The process integrates confidence over time and once the confidence reaches the fixed threshold, the decision is made and the signal is executed. Recent studies, Murphy et al. (2016), Ditterich (2006), Churchland et al. (2008), and Saaty (2007) have shown that the time dependency of the decision making process and the urgency of signals are invoked by humans. These findings show that humans may make decisions with different levels of confidence based on urgency. The more urgent the decision, the less confidence may be accepted. This urgency-based process allows humans to adopt time-variant pressure to execute actions (execution pressure) as a time-variant variable. The first study also showed the existence of neural gain modulation for urgency generation in humans, which implies the existence of a modulation signals. These signals are initiated to express urgency and modulate the confidence level.

Inspired by the biological evidence of the time-variant decision making processes, we propose a model for the multi-sensory decision-making process that recruits a time-variant decision-making signal. The model performs four main tasks. The first one is tracking the changes in the working memory to detect the state change of the stimulation. The second task is threshold manipulation based on the urgency. This second process is the main element which addresses the time-variant feature of the decision making block. The third task is analysing the relevance of the received spatial location within a predefined task by the experimenter. The experimenter should define the relevant working area and the required information to perform the projection. The last task is sending the action execution signal to the action execution block based on the required actions which are also defined by the experimenter. These tasks are defined within three parallel processes. The first process aims to respond to the signal coming from the audio visual perception block that shows the presence of the stimulation and that the urgency of taking a decision should start. The second process is to predict the spatial location of the source of the stimulation from the 2D response of the audio visual perception. Finally, the third one is evaluating the relevance of this stimulation based on its 3D location. The first process works as the urgency trigger which starts a modulation signal for the threshold value of the confidence level for the localisation task. Once the confidence exceeds the threshold, and based on the defined task, the evaluation of the signal starts. If it is relevant to the task then the action is executed.

##### 2.1.2.1. Decision Making Controller

The decision-making controller block is the module responsible to control the flow of decisions, manipulate the threshold of the confidence level in the attention manager, analyse the salient perception output based on the context and finally send the request to the action execution system. The control flow consists of two parallel processes. Each process has events that trigger behaviours. The aims of the first process is receiving the salient hot point from the attention manager, analysing the relevance of this point based on the task information, and finally sending action execution commands if it fulfills the action requirements. The second process is responsible for the control flow and manipulation of the threshold. The following part will explain the events and the behaviours for both of the processes.

In the first process, we have three events that set and reset behaviours. The first event is a trigger event from the audio stimulation. This event sets the decreasing threshold behaviour which sends commands to the attention manager to subtract a defined decreasing rate from the current threshold value. This signal is an urgency signal to the perception block. The second event is the action execution. If the action is executed, the action state in the working memory is set and the decreasing threshold behaviour is reset. The final event is the off trigger of the stimulation. This event sends a resetting signal to the attention manager to reset the threshold and to the action execution block to return the robot to the home position. The resetting signals have two different delays. The threshold reset signal is sent after 0.5 s after the off trigger of the stimulation. The home reset signal is sent after 4 s from the off trigger of the stimulation. These delays are chosen to maintain the stability of the system. The setting and resetting flags for the action, thresholds, and the stimulation are saved and recalled in the working memory which will be explained in the next section.

In the second process, there is only one event, which is receiving a salient hot point under the condition of the idle state. This event starts the evaluation of this point in the task context. The evaluation is the relevance of the 3D projection of this point to the predefined working area in the environment. Knowing the 2D coordinates of the hot point received from the attention manager and the equation of the plane of the working area, we calculate the 3D location in the environment. Based on the defined task, the decision is made whether to do the action or not, and which action to do based on the projected 3D location of the hot point. If the action is done then the action execution event is triggered.

As explained here the processes are parallel. However, they are interconnected, and both are dependent on each other. So the second process is only running when the robot is in the idle mode and the mode of the robot is controlled by the second process. And in the first process, there is a behaviour that is triggered by the second process which is action execution when the mode is changed by the second process. Following the assumption of ignoring the vertical component in the audio stimulation, we implement a function to force the vertical component of the 2D hot point to meet the location of the stimulation sources. This is done by estimating the vertical component given the current head altitude angle and the vertical field of vision. The robot identifies the stimulation source by calculating the distances between the projected 3D location and all the stimulation source. The source corresponding to the minimum distance is the winning location. Finally, the decision-making block sends an action execution command with information about the localised stimulation source to execute an action.

There is stored information related to the task and environment. This means that in this block, the task is defined with its requirement. The task is a defined action under a certain stimulation condition. The task related information is information about the stimulation conditions, the starting level of confidence of the stimulation, the modulation rate which defines the urgency-accuracy trade off, and finally the required action when the conditions are applied. On the other hand, the environment related information in the action execution layer is a higher level information. It includes the locations of the relevant stimulation sources, the working plane, and the action execution parameters. This information helps the robot to project the action from the 2D egocentric frame of the vision to the 3D world and execute it in a proper way. More information related to this section will be explained in the experimental setup section of the paper.

#### 2.1.3. Working Memory

The concept of working memory has emerged in psychology literature as a broad set of mechanisms that explain this accumulation of perceptual information over time. Psychology researchers have shown the relationship between attention and working memory (Schweizer and Moosbrugger, 2004; Phillips and Noelle, 2005). They have shown the irreplaceable role of working memory in solving cognitive problems by maintaining some essential information for certain tasks that involve monitoring the environment. Based on this information, we added a working memory element in our model to endow the robot with this ability. The working memory in our model maintains essential environmental and internal states for understanding the current scenario and for executing the correct action in the defined task. In our working memory model there are two main memory components. The first one is the stimulation states and the second one is the actions states. The stimulation states define whether the stimulation is currently on or off and track it, whereas the actions states define whether the robot is executing the action or has finished the execution or still hasn't executed it for the current active stimulation.

As shown in the Figure 2 the working memory block is bidirectionally connected to both the decision making and perception components. In our implementation, we developed a state working memory. It stores the states of the stimulation, action, and confidence level to enable better interaction with the environment. The stimulation states define whether the stimulation is currently on or off and track it (for both vision and audio). The audio stimulation state is set based on the audio trigger, while the visual stimulation state is defined by the gamma value of the scene. If the gamma value exceeds the threshold, there is a visual stimulation. The attention manager block is responsible for maintaining the stimulation state. Whereas the actions states define whether the robot is executing the action or has finished the execution or still hasn't executed it for the current active stimulation. The decision making controller maintains the state of the action execution as well as the confidence threshold. The attention manager and the decision making blocks are recalling these states in their processes. The working memory block ensures a stable robotic behaviour for attention, decision making, and action execution cycle.

Another aspect of the working memory system is the habituation process. It is a perceptual stage necessary for the humanoid robot iCub to memorise the specific conditions of the environment, as well as details about the human partner. Habituation is a well-studied process in psychology and neuroscience. It is the simplest form of learning (Rankin et al., 2009). It is defined as the process of learning how to filter out irrelevant stimulation and focus only on the important stimulation. (Groves and Thompson, 1970; Wagner, 1979). It is an important biological process for an effective learning. In this work we implement a simple form of habituation which allows the robot to learn the baseline sensorial characteristics of the environment and of the human partner in order to properly compensate during the task.

From the implementation point of view, the cognitive architecture comprises of a habituation signal that is sent to the decision-making block. This signal changes the current task to calculate some parameters from the scene in a defined time period. This signal also informs the process that the stimulation will be presented, and it is required to see the effect of this stimulation and memorise it. When this signal is received, the decision-making block starts to analyse the scene and records the changes. More specifically the Γ value changes. After the defined time period for the habituation process, the initial threshold of the confidence is set by the maximum Γ value during the habituation process, minus a fixed value as a sensitivity zone. The initial threshold value is one of the relevant details in the human robot collaboration with the human partner. In particular, this threshold changes based on the visual environment, which includes the presence of the human subject.

#### 2.1.4. Action Execution

The action execution block receives commands from the decision making block and then executes these commands by performing whole-body motor execution of a required action. The action is previously learned by the robot. The motor action execution is expecting an allocentric location in the working environment. By providing a reasonable assumption about the task, its context, and working area, we were able to define the attentive plane in a geometrical representation. Applying projection on this plane we estimate the allocentric representation of the required point. Based on the task, we assess the spatial relevance of this point and check if this point relies on the predefined working area of the current task. The implemented module for the action execution is called attention action linker.

##### 2.1.4.1. Attention Action Linker

The attention action linker interprets the decision and executes the motor commands. The decision-making layer gives the command to the action execution layer with the result of the decision task. The linker also controls the motor action by enabling or by disabling it. The actions are predefined in the current task. In corresponding to the stimulation source there are two actions, the gaze action, and the point action. This part of the architecture is more task oriented. In this module, the response actions of the robot are defined based on the stimulus location. The main goal of putting this module in the architecture is to enable taking actions after finishing the perception process and making an attentional decision. In the Experimental part we will talk about the Implemented actions for the defined task in the experiment.

#### 2.1.5. Incremental Approach

To sum up, our main contribution is the integration of: perceptual processes, working memory and its rule in attention, time-variant decision making, and finally the action execution into a complete cognitive architecture. Delving deeper into the details, the audio-visual perception has four main contributions. The first one is adding new modules on the top of the audio Bayesian localisation model to create an audio salient based allocentric attention representation. Secondly, the multi-sensory integration, by embedding the audio saliency map as another feature map in the linear combination of the PROVISION model. The third contribution, is the implementation of the asynchronous selection of the saliency. The last contribution in the perception block is the integration of the prior knowledge into the audio attention component to improve the localisation abilities of the robot.

For the decision making block, our contribution relies on the computational implementation of the time-variant threshold manipulation which addresses the confidence-urgency trade off in perceptual decisions. Our final contribution is the integration of working memory in the cognitive architecture, which is inspired by human cognition.

### 2.2. The Experimental Setup

**Figure 3:**
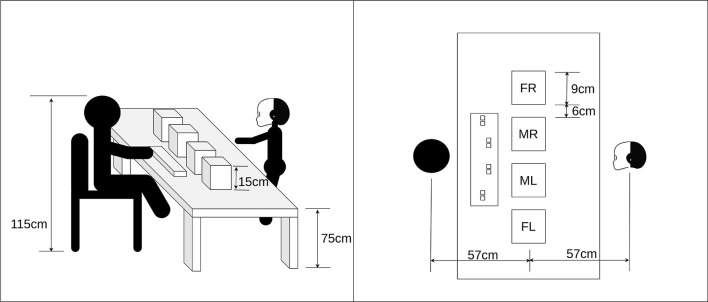
Experiment setup showing the positioning of the robot and the participant. Also, the four stimulation boxes and their locations. Far left “FL,” Middle left “ML,” Middle right “MR,” and Far right “FR.”

We test our hypothesis by performing a joint human-robot attentional task in an unstructured environment. The rationale behind the design of this experiment is the facilitation of the decision making process evaluation, the performance of the system in different stimulation modes (audio-visual vs. audio only), and finally, the comparison between human and robot performances. Figure 3 shows the experimental setup. The robot is facing the human participant. In between, there is a table that has the stimulation board and a keyboard in front of the human participant. The stimulation board is approximately centralised between the robot and the human with 57 centimeters distance to both. The height of the chair where the participant sits is configured so that the human is on the same level as the robot. This height places the stimulation board within an optimal location for the field of vision for both the robot and the participant.

#### 2.2.1. Participants

We conducted the experiment with 21 healthy participants (female: 14, male: 9) aged between 26 and 43 years old, with an average age equal to 30.5 ± 4. All participants voluntarily participated and signed an ethical and information consent approved by an ethical committee at San Martino Hospital in Genoa, Italy. All the participants work within the institution with no direct involvement to the research.

#### 2.2.2. Stimulation

We built a stimulation setup which consists of four identical boxes. The boxes are placed horizontally on the same line. We noted the names of the boxes with respect to the robot's frame of reference: (FL) for the far left box, (ML) for the middle left box, (MR) for the middle right box, and (FR) for the far right box. Each box can produce both audio stimuli and visual stimuli. The visual stimuli are produced by a smart bulb. The smart bulb emits up to 800 luminous flux. We use red colour with the maximum luminous. The audio stimuli are produced by a three watt Bluetooth speaker. Both the bulb and speakers are embedded inside the box. The top layer of each box has holes where the light and sound waves can prppagate through, but that hide the smart bulb. The width of the box is 9 cm. The boxes are placed with 15 centimeter separation distance (center to center). Therefore, the distance that separates the boxes is 6 cm. We placed the stimulation boxes in this configuration with the given spacing to make sure that all boxes are within the direct field of view (the view with a zero yaw angle for the face) of both the robot and the human participant. Additionally, we made the task more challenging by minimising the distance between the boxes. As it is proven that human perception matches sound sources and visual sources for angles as large as 30 degrees apart (Jack and Thurlow, 1973). we selected a long distance as half of 30 degrees and a short distance as one fourth of these 30 degrees. This drove our choice for the configuration setup. We use a complex tone with a 1 KHz fundamental frequency and 3 harmonics for audio stimulation. The visual stimuli is a red light emitted from a smart bulb. The choice of the complex frequency and the red colour is because of their high saliency compared to other colours for the vision, and simple tone for the audio. This was chosen to ease detection for both the human and robot.

#### 2.2.3. Task Description

The task for both the human and the robot is to identify the active stimulation box and react as quickly as possible. There are two types of activation for the stimulation boxes. The first type is audio only stimuli and the second type is audio-visual stimuli. Only one box can be activated at a time. The stimuli are activated for a fixed time (10 s). The time between rounds is also fixed at 10 s. The stimulation trials were distributed equally over the four boxes. So, each box was turned on 25% of all trials. Also, the stimulation types were distributed equally. 50% of the trials were auditory-only and the other 50% are audio-visual. Each box was activated for 8 trials, 4 of them were audio-only and the other 4 were audio-visual. The sequence of trials and the type of stimulation were randomised, but fixed across participants.

In the implementation section, we mentioned that the user defines the task for the robot and gives to the system the required information for the task and its environment. Therefore, we defined the task on the top of the attention system. The task is to localise the stimulation from a set of defined sources located horizontally in front of the robot. After localising the location, the robot should execute gaze action (to look to the stimulation source) and point action (to point with the arms to the stimulation source. We provided the robot with the environment related info which are the working plane where the stimulation sources are located, and the working area on this plane. Additionally, we informed the robot that the stimulation sources are in that defined area in space. Consequently any localised stimulation within this area is considered as relevant to the task. If the localised stimulation is outside this area, then the robot ignores it as it is irrelevant stimulation. Extra environment information was added to the robot here, including the stimulation sources count and location. After localising the 3D location of stimulation, the robot should identify the source of this stimulation from the defined set of sources. To sum up, the task is stimulation localisation which is estimated in the decision making layer. This task divided into 2 stages, the first stage is localising the stimulation within the 2D frame and the second stage is to check the relevance of this stimulation when the 2D location is projected into the 3D world. If it is relevant, then the robot will execute the action. The next section is describing the defined actions for the robot and also for the human participant.

#### 2.2.4. Human/Robot Reaction

We placed a keyboard in front of the human participant. On this keyboard, eight buttons were highlighted in four groups. Each group consisted of two side by side buttons. The human participants were requested to react as fast as possible by pressing any of the two buttons within the buttons group, which correlated to the activated stimulation box. We decided to use two buttons in the keyboard to increase the pressing area in order to simplify the action and minimise the execution time. On the other hand, we defined two actions associated with each localised stimulation box. The first action is a pointing action using the arm, the hand, and the fingers while the second action is a gaze action using the head and the cameras (eyes) of the robot. For the right side boxes the robot will point to the selected box (FR,MR) using its right hand. Similarly, the left hand is used for the left side boxes (FL,ML). For the gaze action, movements in head and the cameras are involved. The reaching action is biological and human-like movement that recruits not only the entire upper body of the humanoid robot iCub, but also the control of head and gaze of the robot. The gaze action brings the fixation point (line of sight) on the target with optimal coordination of the 6 degrees of freedom of head and eyes. The pointing with the index finger of the most opportune hand brings the robot to assume a new posture in less than 2 s. The coordination between head movement and upper body movement is designed in detail and makes the whole body movement look natural and human-like. It is possible that the human participant‘s attention is biased by this movement, but this is useful information in order to estimate the human-robot mutual influence in joint tasks.

#### 2.2.5. Measurements and Rounds

The robot and the human do the task together at the same time. Before the first trial for each subject, we introduced the visual stimulation for the robot and the human. The robot performed the habituation process with the starting signal during this stimulation introduction period. Our first aim was comparing the performance of the robot vs. the performance of the human participants in terms of both accuracy and reaction time. In general, we were also interested in measuring how much one participant influences the other in human-robot collaboration. In order to measure accuracy and reaction time for the human participant we recorded the pressed keys and their correspondence to the target as well as the reaction time. For the robot accuracy and reaction time, we recorded the action execution commands of the robot and the internal triggering commands of these actions as relevant information about the timing and selected location. Additionally, we aimed to analyse all components of the decision making processes. Thus, we recorded the threshold profile (indicating the urgency to act) as well as the integrated scene analysis which includes the Γ value (indicating the confidence on target localisation process) during the whole trial. The second aim was to understand the behaviour of the human participants considering the presence of the robot. Specifically, in this experiment we focused on gaze behaviour. We recorded the gaze data during the whole experiment using Tobii pro glasses. This data includes the 2D gaze location within the field of view of the camera and the gaze event (Fixation/Saccade). This is the main data from the eye tracker that we focused on. For better analysis we developed a program to ensure synchronisation between the eye tracker time stamp and the time stamp from our system. The idea of the program is to send a timestamp instance from our system to the Tobii pro glasses, and in the analysis stage we map the timestamp of the eye tracker to our system's timestamp. The synchronisation process ensures the transfer of the trials' information to the gaze data. The trials' information mainly include the current state of the stimulation, the active box, the starting time of the trial, and the type of the stimulation.

## 3. Results

We primarily focused on assessment of the performance of the memory-based cognitive architecture for joint attention. To perform an extensive evaluation of the system, we subdivided the analysis into two main sections. The first section is analysis related exclusively to the performance of the cognitive architecture. This includes the evaluation of the whole system dynamics which is mainly the decision making process and the overall performance (localisation accuracy and reaction time) by comparing it with human performance in a similar attentive challenge. The second parts of the results is a detailed analysis of the gaze patterns. Given a thorough description of how the focus of attention was jointly redeployed, we focused our secondary analysis on the gaze patterns of both the robot and human participants. Such gaze behaviours are a direct result of attentional processing but more importantly tend to cause mutual influence between the robot and human. Humans tend to look where their partner directs their gaze (Frischen et al., 2007). Also, it is an important component in joint attention (Yu and Smith, 2013). So, the actions of the robot which are the gaze movement and pointing might influence the attention of the human toward a specific location. On the other hand, the gaze action of the human changes the visual features of the scene while the head moves. Consequently, this creates changes in the saliency map of the robot which might change its behaviour, and this what we want to analyse.

### 3.1. The Performance Analysis

#### 3.1.1. The Memory Based Decision Making Process

We evaluated the memory-based decision-making process to report how the cognitive architecture makes the decision to act, averaged across all trials. The process is based on working memory, the confidence measure and the decision threshold (the threshold in which if the confidence reached, the agent will make a decision) as core factors of the decision-making process. The cognitive system makes the decision to act in presence of the event of crossing between the confidence measure and the threshold curve. Therefore, we analysed the decision-making behaviour to assess the effect of working memory as well as the performance of the confidence measure and the decision threshold, which are core factors of the decision-making process.

Adding working memory allowed the robot to track the stimulation state of the trial (presence of a stimulation), and the state of his own action (whether the action is done, or in progress, or not yet executed). This has a clear advantage with respect other work done in the recent past (Gonzalez-Billandon et al., 2019). Once the robot executed an action for a certain stimulus, it could realise that the task is done and there is no need to execute the action again until the current stimulus stops. This represents its internal working memory of the active motor actions. When the stimulus stops the working memory is updated, allowing the robot to reset and wait for another stimulus. Thanks to this mechanism the robot was successfully able to execute the action on the right time frame (after the stimulus turned on and before it turned off) in 95.8% of the trials.The working memory stabilises the action cycle and also allows the execution of the action based on meaningful environmental and internal states. This leads us to accept the first hypothesis, “The memory-based cognitive architecture is able to attend to multi-sensory stimulation and correctly make a decision based on the localisation process.”

**Figure 4:**
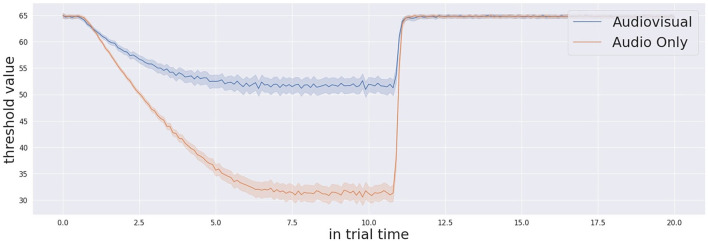
Confidence threshold profile across the whole trial time (20 s) for both audio-visual and audio only trials.

**Figure 5:**
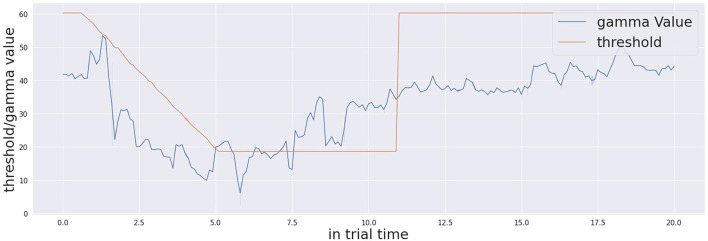
Gamma measure (Ŵ) and the threshold profiles in one of the trials across the whole trial time (20 s). The crossing occurs around 5 s.

**Figure 6:**
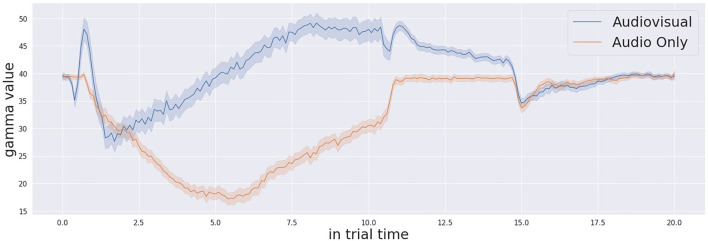
Confidence profile (gamma measure Γ) across the whole trial time (20 s) for both audio-visual and audio only trials.

Moving to the analysis of the confidence measure and the threshold manipulation, Figure 4 shows the average threshold profile with audio-only trials in blue and audio-visual trials in orange. The initial threshold is different for each participant. This is due to the habituation process, as the system memorises a different initial threshold for each participant. The process runs at the beginning of the experiment for each participant, because this initial threshold is dependent on the visual features of the environment including the human participant in the field of view. Thanks to the working memory, the robot retains important information of its task and this contextualisation is not only related to the environment. The starting time of the threshold modulation process is based on detecting the existence of the stimulation. Thus, the exact starting time of the modulation signal is different from one trial to another. Similarly, the confidence incremental process defines the action execution time together with the threshold decision. Therefore, the linear decreasing rate creates a curved, averaged response. After execution of the action, the decision making process slows down the threshold decreasing rate and this creates the flat part of the curve observed in Figure 5. In the audio-visual condition, the threshold decreasing rate slows down earlier. This is because the action is typically executed earlier due to the greater level of confidence in target localisation. After the multi-sensory stimulation stops (experimentally fixed in time after 10 s from the beginning of the stimulation), the threshold resets again to the initial value. In this exact moment, in the audio-only trials, the threshold starts from a lower value. This reflects the lower confidence and consequently the longer response time to take a decision to act. On the other hand, by looking at the Γ measure in Figure 6, we observe that the Γ function in audio-visual trials (orange curve) produces a spike almost instantaneously after the beginning of the stimulation. This is due to the visual saliency of the stimulation, which provides a strong, unique visual stimulation in the field of view. In the audio-only trials the Γ function shows that the confidence decreases at the beginning as causal effect of proactive sensing (the robot tries to eliminate the effect of the environment noise) and it starts to increase (after approximately 6 s in average) till the stimulation ends. When the threshold profile and the Γ measure cross one each other, the cognitive system makes a decision that triggers the action of pointing to the target stimulus.

**Figure 7:**
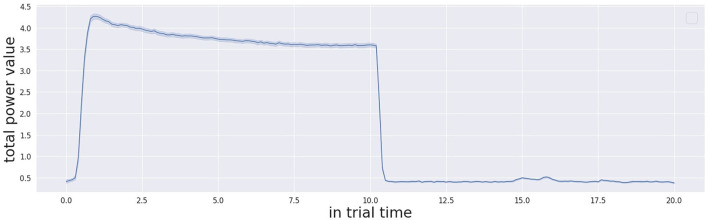
Audio power profile across the whole trial time (20 s) during all the trials.

It is also important to describe the decision-making process in detail by presenting an example trial. Figure 5 shows a single trial taken from one participant. Once the simulation starts, the threshold of confidence starts to decrease in time with a decreasing factor from the initial value (the parameter is specific to the participant, computed during calibration, and kept in memory by the system). The level of confidence indicated by the Γ function and the threshold profile progresses in time under their proper temporal dynamics until the Γ value and the threshold cross each other. At this point, the cognitive architecture makes a decision and acts, by consequently pointing to the estimated source of stimulation. Once the stimulation ends (after 10 s from its beginning) the system waits 0.5 s and then resets the threshold to the initial value. The starting and stopping of the trial stimulation are autonomously detected by system based on the audio power in the audio signals received by both the microphones as presented here in Figure 7. The reset of the threshold profile to the original value occurs exactly 0.5 seconds after the end of stimulation is detected.

#### 3.1.2. The Overall Performance (Accuracy and Reaction Time)

**Figure 8:**
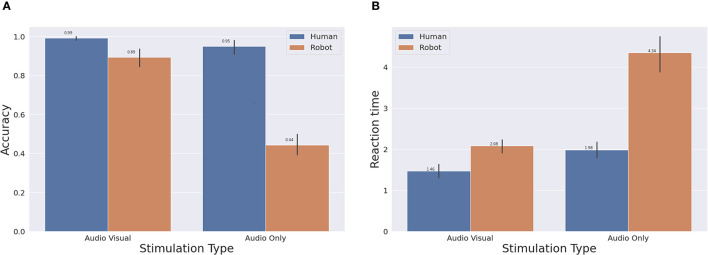
Overall performance across the different types of stimulation for both the robot and the human participants. **(A)** Accuracy. **(B)** Reaction time.

To assess the performance of the robot, we compared the attention system of the robot with human performance in response to the same multi-sensory stimulation and mutual sensorial influence. We analysed the overall performance based on (a) the reaction time and (b) accuracy as the primary source of evaluation. In particular, we characterised the performance based on the two stimulus typologies: audio-only stimulus or audio-visual stimulus. Figure 8 shows the measure of the reaction time and accuracy for both the robot and the human participants, averaged across all the trials/participants. The bars in orange indicate the performance of the robot and the blue bars indicate the performance of the human participant. The participant and robot‘s choice is considered wrong if the identified box wasn't the active box or if the action didn't execute. Looking into the accuracy for each of the stimulus types separately, the robot records similar performance to the human in audio-visual attention tasks. The robot autonomously identified the source of the stimulation with 89% average accuracy. On the other hand, the robot performed with 43% average accuracy in the audio-only trials. The audio-only trials were more challenging for humans as well. To assess performance, we performed multiple t-tests to compare the behaviour of the human in the audio-visual task vs. audio only task, and similarly for the robot. The results of all the tests that demonstrated significant differences are the following:

- Human audio-visual reaction time vs. human audio only reaction time : *t*_(40)_ = −3.7527, *p* < 0.01.
- Human audio-visual accuracy vs. audio only accuracy: *t*_(40)_ = 2.1436, *p* = 0.0382.
- Robot audio-visual reaction time vs. robot audio only reaction time: *t*_(40)_ = −9.6, *p* < 0.01.
- Robot audio-visual accuracy vs. robot audio only accuracy: *t*_(40)_ = 12.2, *p* < 0.01.

So there are significant differences of both reaction time and accuracy between the audio-visual condition and audio only condition for the human participants and also for the robot. The differences in the case of the robot were all significant (*p* < 0.01). As the average accuracy value for audio-visual is higher, and the reaction time is lower compared to the audio only task (shown in Figure 8), we accept our second hypothesis that “The stimulus localisation accuracy and reaction time of the robot in the audio-visual task is better than in the audio only tasks.”

We also performed t tests to compare the performance (reaction time and accuracy) of the robot vs. the performance of the human in the localisation task. The statistical tests showed significant differences between both performances as follows:

- Human audio-visual reaction time vs. Robot audio-visual reaction: *t*_(40)_ = −4.99, *p* < 0.01.
- Human audio-visual accuracy vs. Robot audio-visual accuracy : *t*_(40)_ = 4.06, *p* < 0.01.
- Human audio only reaction time vs. robot audio only reaction time: *t*_(40)_ = −9.7, *p* < 0.01.
- Human audio only accuracy vs. robot audio only accuracy: *t*_(40)_ = 14.9, *p* < 0.01.

Thus, we reject our third hypotheses The performance (accuracy and reaction time) of the robot will be as good as the performance of the human participants in localising the stimulus.

We did further statistical investigations using Wilcoxon signed ranked test (Rey and Neuhäuser, 2011) to test how different the performance of the robot was compared to the human. We found that the accuracy drop in the audio-visual condition is statistically less than 20% of the human accuracy. Also, the difference in reaction time of the robot in the audio-visual condition compared to the reaction time of the human is less than 1 s which is 70% of the increase in human reaction time. For the audio only condition, the difference was much bigger than the audio-visual condition. The differences in the audio-visual condition are comparable considering the complexity of the system and processing speed of the machine. The audio only condition is more complex compared to the audio-visual condition for both the human and the robot. However, the complexity of the audio-only localisation task does not entirely explain the considerable gap. To understand the reasons of this performance drop, we more thorougly investigated the conditions of wrong actions. The results are shown in Table 1. There are two conditions in which we consider the behaviour of the robot to be worse. The first condition is when the action is executed but the identification of the active box was wrong and is annotated with (wrong identification). The second condition occurs if the action never executed during the on time of the trial and we annotate this behaviour as (no action). For the human, all the wrong action trials were due to wrong identification. For the robot, the first condition occurred most of the time (89% of the total failures). On the other hand, there were two causes for no action failures. The first cause is when the robot executes an action in the off time of the stimulation due to some confusion from visual features in the scene. More specifically, it was observed that for some participants the robot got confused from the hand of the participant, indicating once again how mutual influence impacts attentive tasks. The hand worked as visual stimulation and the robot identified the closest box to the hand as a source of stimulation during the off time. If the robot executed an action during the off time, the robot does not reset the exception event before the end of stimulation of the next trial. The consequence of this is a (no action) failure for the trial next to the off time when the robot executed the action. This actually happened very few times (15 times) across all trials, which consists of 6% of the total failures. This is 60% of the second type of robot failures (No action failure). The remaining 40% of the no action failures are due to low confidence level. The robot did not execute an action few times because the confidence value (Γ value) never reached the threshold during the on time of the trial. This type of failure only forms 4% of the total failures.

**Table 1:** Robot's failure types percentages.

	**Failure**		**Percentage from total failures (%)**	
Type 1	Wrong identification		89.4	
Type 2	No action (Wrong action type in previous trial)	60%	6.4	
	No action (low confidence)	40%	4.2	

Based on these analyses, the major cause of failure is wrong identification. Therefore, it is also important to analyse in detail the attentive process in time. More specifically, the audio components need to be analyzed, because the difference in performance lies in the temporal response of the attention system. So, in the next section we analyse the temporal responses of the audio probabilities, which are the base of the localisation process during the audio only condition.

#### 3.1.3. Detailed Analysis of the Audio-Only Trials (Probability Profile)

Since the behaviour of the decision making process does not show erroneous behaviour, but instead the decision is made in the right time frame with a reasonable level of confidence, we believe that the reason for the worse performance in audio-only trials is to be found in the localisation process. As shown by a more detailed analysis for audio-only trials, the localisation process is based on the level of confidence that each box is the target, in other words the probability that each one of the four locations is the target. Such probability changes over time for each potential location of a stimulation source. In the audio-only condition, the probability profile is extracted from the Bayesian map, which is the output of the audio localisation system. The temporally detailed analysis of the probability profile is carried out during the 20 s time frame of the trials. During the first 10 s, the auditory stimulation is generated by the target box only.

**Figure 9:**
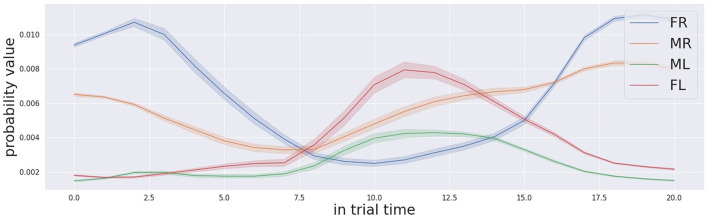
Audio probabilities profile for the trials that the far left box (FL in red colour) was activated.

**Figure 10:**
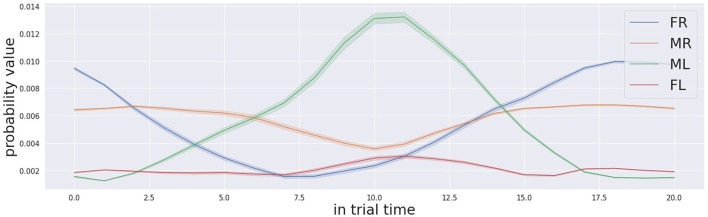
Audio probabilities profile for the trials that the middle left box (ML in green colour) was activated.

Figures 9, 10 show the probability profiles for the 4 locations of the stimulation sources when the active box is the far left one and the middle left one respectively. The response is averaged across all trials. The first relevant point of these figure is that the shape of the curves are similar for the boxes located on the same side, independently from the location of the source of stimulation. In other words the probability profile over time of the far-right is similar to the one middle right and similarly the probability profile of the far left is similar to the middle left. Such results indicate that there are differences for time progression of the probability profile between the left and right boxes from the location where the robot is standing. Such difference has an impact on the localisation of the sound target since the certainty of sound location changes over time differently between the left and right boxes. Similar difficulty from one side over the other was actually reported by most of the participants. Another aspect that might have an impact on the localisation of the source of sound is that the probability profile of the sound sources from the same side evolve similarly. This makes the discrimination task complex for the robot, but also for the human participant. It was challenging for them to identify which box between the 2 boxes in the same side is the stimulation source in audio-only trials. The similarity between human robot participant in same-side during sound discrimination suggests that the Bayesian modelling implemented in the cognitive architecture shares some similarities with human behaviour.

Another relevant point relates to the temporal profile of the probabilities for the different salient locations. The probability corresponding to the right location increases with time as long as the stimulus is active, (in the first 10 s) which is the right and required behaviour. However, the probabilities of corresponding matches between the source of sound and different locations do not always start from zero and equal values. This indicates that before the activation of the stimulation, the localisation system believes that one location is more likely to produce sound than another location. Each probability goes to an initial value that is not equal to zero and also not equal to other location's probabilities. Our speculation explains the presence of these two phenomena as the result of acoustic noise in the environment. The acoustic noise equally affects the performance of the robot and of the human participant. It would be wise to remove the constant acoustic noise in the environment to eliminate its effect on the Bayesian map probabilities first, and then integrate evidence from the actual stimulation over time.

The final consideration regards the time the system requires to make the right decision. From both graphs, we observe that it takes in approximately 7.5 s for the far left box to be the box with the highest probability and 6.5 s for the middle left box. For the boxes located on the right side of the robot, the value for the middle right is similar and is approximately 7 s. For the far left box, the system struggles due to the noise, the probability for the far left never reaches the maximum when the box was activated within the on time frame (10 s). The decision-making process is tuned with some parameters to react faster than the required time. So the average reaction time of the robot for audio-only stimulus was measured to be around 4.34 s (STD: 1 s), definitely faster than the time necessary for the temporal probability profile to converge on the correct stimulation. Thus, we note that the attentive system can localise the target with a higher accuracy if the decision making process is allowed a longer reaction time. However given enough time, the auditory localisation process is always correct and the probability of the correct target always exceeds the probability of the others. For example, the audio probability profile for the far left box is the highest after 7.5 s. For the middle left box the audio probability profile for the middle left target is the highest after 6.5 s. Such fine refinement is actually doable in the cognitive architecture proposed, since by adjusting the tuning parameter we can refine the decision-making process and adjust the decreasing rate of the threshold.

**Figure 11:**
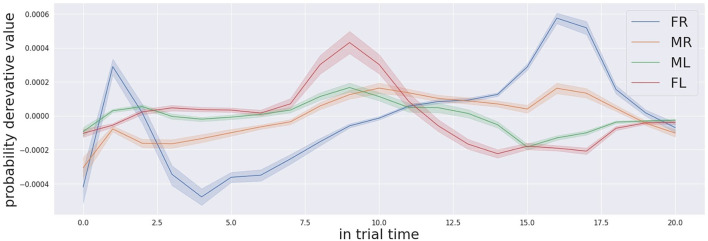
Derivative of the audio probabilities profile for the trials that the far left box (FL in red colour) was activated.

**Figure 12:**
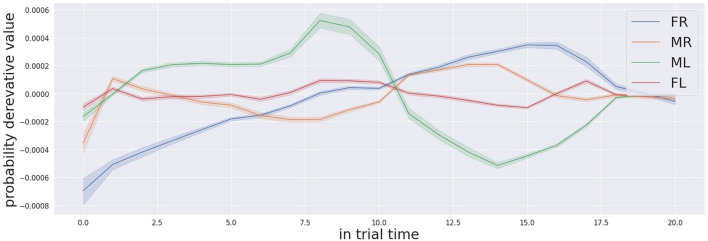
The derivative of the audio probabilities profile for the trials that the middle left box (ML in green colour) was activated.

In conclusion, we assessed that the task results are also difficult for the human participants according to an interview in the debriefing phase of the experiment. Another relevant observation in regards to the numerous comments of many participants indicates the change in the auditory landscape as the most meaningful cue to localise the target. The suggestion convinced us to looked in the change rate of the confidence level for the different possible targets. In the Figures 11, 12, we show the change rate of the confidence probabilities for the four locations for the trial respectively when the target is FL and ML. We noticed that the attentive system can localise the target correctly in a shorter time if decision making process analyzes the change rate of the confidence probability instead of the confidence probability. For example, for the target in FL (see Figure 11) the correct detection of the target can occur as early as approximately 3.0 s, and for the target in ML (see Figure 12) the correction detection the target can occur as early as at approximately 2.5s.

### 3.2. The Behavioural Gaze Analysis of the Human and the Robot

The behavioural analysis of the human participants gives us a relevant insight on the mutual influence between the two partners. The behavioural analysis relies on data from the eye tracker. We were able to record the gaze data of the human participants. The gaze data is the 2D location of the gaze and the gaze event. The gaze events can be one of two types: fixation and saccade. We aimed to count the fixation events on the stimulation boxes and also on the robot's face during each trial. So, we had to define where the 2D location is projected in the 3D world. We are interested in 5 regions (the 4 stimulation boxes, the robot's head, and other areas). The eye tracker gives the 2D location of the gaze in the camera frame, which changes when the participant moves their head with respect to the world. In order to cluster the fixation events based on the 2D location into 6 clusters, we had to transfer the 2D location from the camera moving frame to a global fixed frame. We achieved this by extracting a reference point in the scene which always exists and then we track this point. This point works as a reference point and all interested regions are defined with respect to this point.

**Figure 13:**
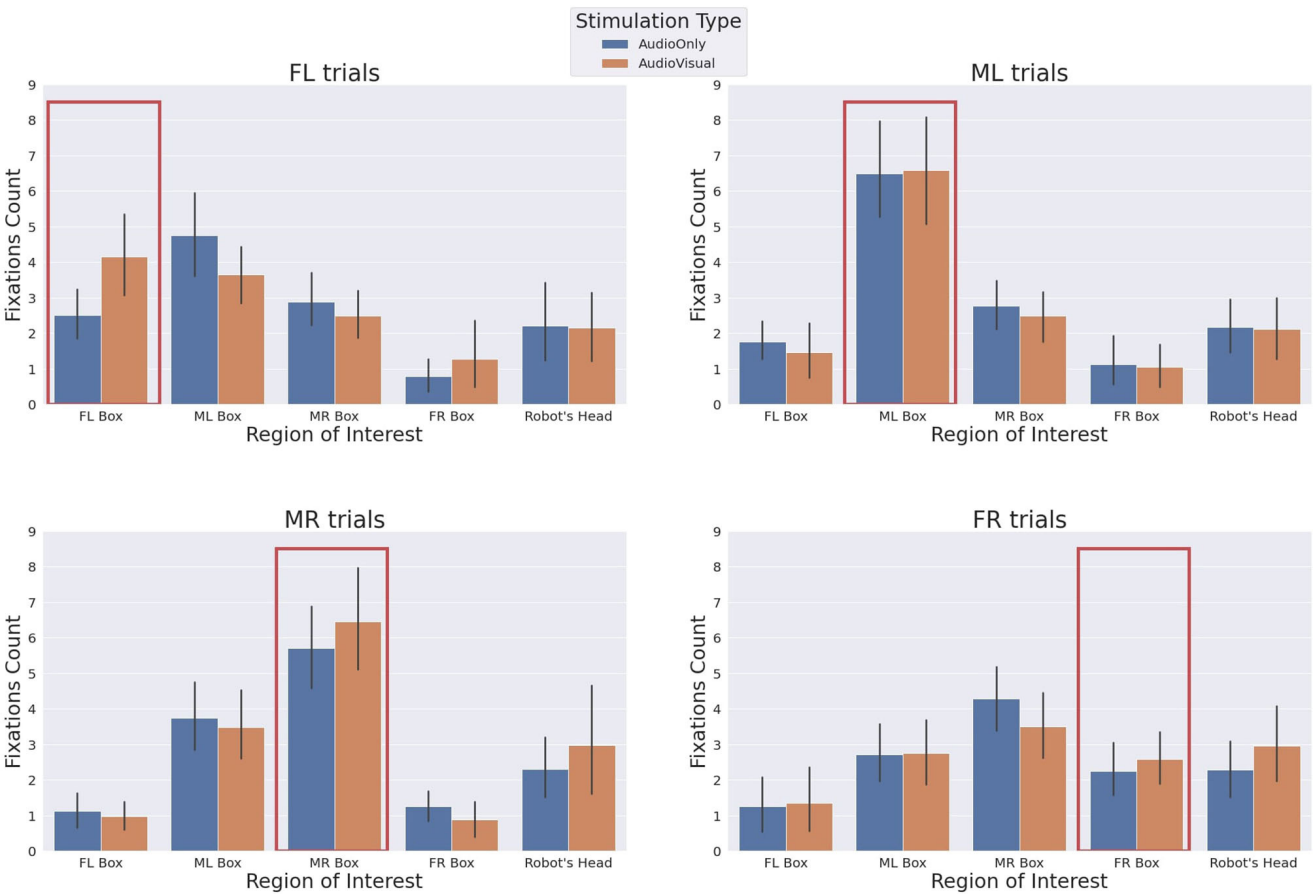
The gaze behaviour of the human.

**Figure 14:**
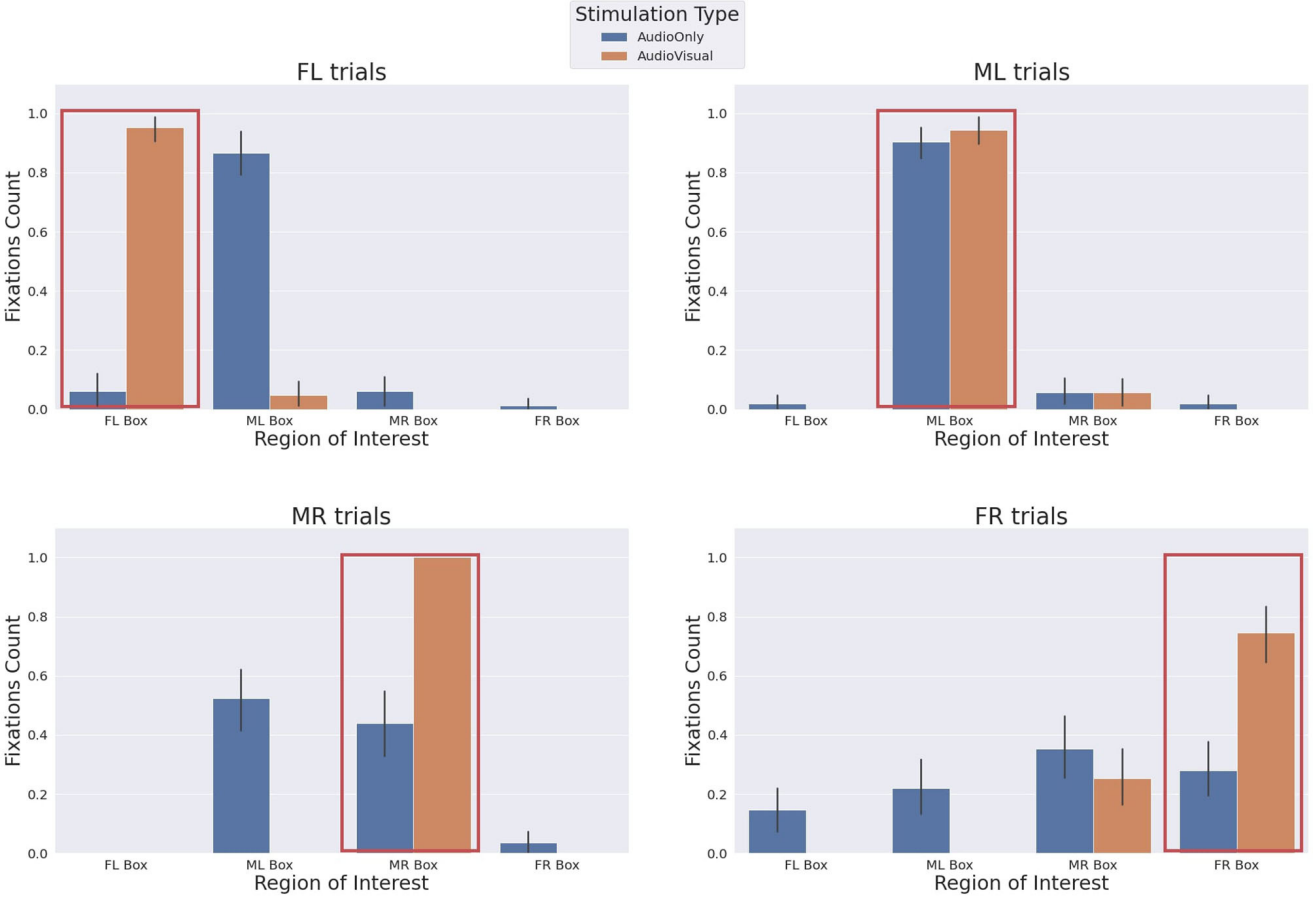
The gaze behaviour of the robot.

From the 21 subjects of the experiment, we could extract the gaze data perfectly from all 12 of them. Three subjects were moving their head very rapidly, and due to this the process of extracting the reference was not accurate enough. The gaze data of 5 participants weren't accurate enough to be considered because the eye tracker failed to calibrate their eyes. So in this section we only consider the data of the 12 subjects for which the calibration was accurate and the reference extraction process was sufficient. The robot behaviour in this experiment consists of its actions, which are the gaze movement toward the selected box and the pointing action with the arm. Figure 13 is showing the fixation distribution in trials. It is divided into 4 panels based on the location of the simulation. (FL, ML, MR, and FR for top left, top right, bottom left, and bottom right panel respectively). The y axis shows the fixation counts. The x axis here is the five defined regions of interest (4 stimulation boxes and the robot's head). We also categorise it based on the stimulation type: audio only in blue and audio-visual in orange. Similarly, Figure 14 shows the gaze of the robot. The robot only does one fixation event during each trial, which is the action of the task. So, the graph also represents the action distribution of the robot. The fixation counts on the active stimulation box is marked with a red rectangle surrounding the bars of this location in each of the panels for both the robot and the human participants. We divide our findings into two parts. The first part is for audio only trials and the second part is for audio-visual trials.

The first observed information is that in audio-visual trials the participants do fixation events on the active stimulation box more than other boxes in FL, ML, and MR trials. But in trials during which FR box was active, the participants do more fixation events on MR box on average. This drive us toward the second observation. Looking into the robot's gaze behaviour, we found that in the FR trials, the robot was confused toward the MR box and sometimes performs gaze actions toward the MR box instead of FR. The next three observations are in the audio-only trials. In the FL trials the robot mostly was driven toward the ML box. This records the highest average in comparison with the other boxes. Similarly, the participants also do more fixation events on the ML box, even more the correct active box which is FL. The second observation in audio only trials are in the ML trials. In these trials both the robot and the participants do fixation events on the correct box more than other boxes. Thirdly, in MR trials the confusion of the robot was between the right box (MR) and the ML box. But it is less than the confusion in FL trials. On the other hand the participants' gaze record the highest count on the right box (MR) and the second highest is the ML box. Finally, it is clearly shown that the participants also spend time looking to robot's head in all trials for all conditions.

## 4. Discussion

Joint attention is a fundamental component for better collaboration in real-world scenarios, such as in industrial environments where the robot and the human worker have to be aware of the products being manufactured (indicated by machinery through visual and audio features). They will be able to coordinate their actions and activities when initiated through their joint attention directed to the same target. The proposed biologically inspired cognitive framework, based on a multi-sensory attention system and supported by memory, constitutes the computational model used to evaluate emergent joint attention between the human participant and the artificial agent. The study had three main hypotheses. H1-Memory-based Decision Making process: The memory-based cognitive architecture is able to attend to multi-sensory stimulation and correctly make a decision based on the localisation process, H2-Audio-visual vs. Audio-only: The stimulus localisation accuracy and reaction time of the robot in the audio visual condition will perform better than in the audio only condition. H3-Robot performance: The performance of the robot will be as good as the performance of human participants. To answer the hypothesis we designed a multi-sensory task, and presented the task to the human participant and the robot. The setup includes stimulation boxes, which are a general model for real-world applications. Thus, we were able to compare the performance of the robot with the performance of a human participant in the same task which is an important aspect defining the quality of the interaction. The comparison focuses on the assessment of both agents in terms of the execution of the same localisation task with the same response time. The rationale behind the co-assessment of both the participants is that we intend to assess the performance of the robot and the human to measure how much they can coordinate in the joint task and to also measure the mutual influence between the robot and the participant.

The statistical analyses resulted on accepting the first two hypotheses (H1-Memory-based decision making process: The memory-based cognitive architecture is able to attend to multi-sensory stimulation and correctly make a decision based on the localisation process and H2-Audio-visual vs. Audio-only: The stimulus localisation accuracy and reaction time of the robot in audio visual task in better than in audio only tasks). and rejecting the last one H3-Robot performance: The performance (accuracy and reaction time) of the robot will be as good as the performance of the human participants in localising the stimulus.

However, further statistical analyses showed that the performance of the robot in the audio-visual condition is comparable, as the accuracy drop was less than 20% of the human accuracy and the reaction time differences were less than 1 s which is less than 170% of the human reaction time. These values are acceptable considering the machine processing speed of such complex computational processes. Indeed the cognitive system is less reactive in audio-only stimulation and only partially influenced in the different internal processes by the presence of the human partner. Although the audio only condition is in general a challenge for both the human and the robot participant, the analysis showed that the main cause of the performance drop in the audio only condition is the false audio localisation, which is caused by the acoustic egocentric noise.

Furthermore, we performed a more detailed analysis of the cognitive processes, and we realised that the decision-making process is robustly designed to swiftly guide the system to make a decision with excessively fast temporal dynamics. On the contrary, the auditory attention system requires longer time periods to make the Bayesian network converge, and thus localise the auditory target. Whereas the auditory localisation process is correct in inferring the location, also in presence of environmental noise (typical in robotic applications), the temporal dynamics of the system require longer periods for the processing of the auditory stimulation. However, the specific inefficiency is of simple resolution for two reasons that we intend to verify in future work. First, the specific modular structure of the developed cognitive architecture and its parametric configuration is designed to allow for fast re-adaptation of the decision-making process. As one possibility, by reducing the urgency to act parameter in the decision-making process, we can allow more time for the Bayesian network to converge, and consequently, we can guarantee improved accuracy. However, although the specific solution improves the accuracy it does not guarantee a faster reaction time. Secondly, thanks to the margin for faster response during auditory localisation, the process allows us to provide more auditory evidence for Bayesian integration in the same time interval. Faster processing of auditory stimulation is expected to improve the reaction time of the auditory localisation system and make it more similar to the reaction time of human participants.

Undoubtedly, the temporal dynamics of how auditory evidence is integrated is a very important aspect. We noticed in human participants that changes in the auditory landscape are more meaningful for target localisation than a static auditory landscape. The same process based on changes in the Bayesian network facilitates the process of inference over the stimuli localisation. The importance of relative changes in the auditory landscape, together with the importance of proactively creating such changes in the auditory landscape (self-programmed head movements) is a promising area of study, and we are planning to exploit it further in future work. Nevertheless, even without these improvements, the cognitive architecture has been demonstrated to be effective, and it shows a natural and robust joint attentive behaviour for Human-Robot interactive tasks. Furthermore, for a thorough understanding of behaviour related to mutual presence and its mutual influence, we also analysed the gaze behaviour of the human participants. The results showed that in the conditions in which the robot confused the location of the active box, the human participants tended to do more fixation events on the wrong box, suggested by the wrong behaviour of the robot. Also, the participants spend time looking at the head of the robot during the experiment, which shows how the human participant and the robot mutually influence each other in similar interactive tasks. This brings us to conclude that the behaviour of the robot may reinforce the gaze of the human toward the robot‘s chosen box. This is reinforced by the robot's behaviour which is both built on the directed gaze and the pointing actions. In the future, we intend to investigate this aspect further with more statistical evidence, and we intend to know whether this hypothesis of the mutual reinforcement is confirmed and what exactly drives it: whether the gaze or the pointing or a combination of both have a stronger effect on the human partner. Finally, we believe that the proposed system paves the way to human-robot collaboration, since coordinated joint attention is proven to facilitate coordination between the interacting parts. Such an optimal mechanism of coordination is considered one of the main facilitation mechanisms in multi-partner interaction tasks. We also showed that the robot affects the gaze behaviour of the participants. Furthermore, with this cognitive architecture, we demonstrate the importance of implementing a complete cognitive architecture (including working memory) in order to attend to salient targets in the environments as humans do. By sharing the same attentional focus redeployment mechanism with the human partner we provide effective joint attention that essentially emerges from environmental stimulation and reinforces natural human-robot collaboration.

## Data Availability Statement

The raw data supporting the conclusions of this article will be made available by the authors, without undue reservation.

## Ethics Statement

The studies involving human participants were reviewed and approved by San Martino Hospital, Genova. The patients/participants provided their written informed consent to participate in this study.

## Author Contributions

OE designed and implemented the modules in the cognitive architecture whereas all authors equally contributed to the design of the cognitive architecture and the writing of the manuscript.

## Conflict of Interest

The authors declare that the research was conducted in the absence of any commercial or financial relationships that could be construed as a potential conflict of interest.

## Publisher's Note

All claims expressed in this article are solely those of the authors and do not necessarily represent those of their affiliated organizations, or those of the publisher, the editors and the reviewers. Any product that may be evaluated in this article, or claim that may be made by its manufacturer, is not guaranteed or endorsed by the publisher.
